# Communicating medication risk to cardiovascular patients in Qatar

**DOI:** 10.1108/IJHCQA-10-2016-0152

**Published:** 2018-02-12

**Authors:** Kerry Wilbur, Arwa Sahal, Dina Elgaily

**Affiliations:** 1 Qatar University, Doha, Qatar; 2 Hamad Medical Corporation, Doha, Qatar

**Keywords:** Patient safety, Education, Medicine

## Abstract

**Purpose:**

Patient safety is gaining prominence in health professional curricula. Patient safety must be complemented by teaching and skill development in practice settings. The purpose of this paper is to explore how experienced pharmacists identify, prioritize and communicate adverse drug effects to patients.

**Design/methodology/approach:**

A focus group discussion was conducted with cardiology pharmacy specialists working in a Doha hospital, Qatar. The topic guide sought to explore participants’ views, experiences and approaches to educating patients regarding specific cardiovascular therapy safety and tolerability. Discussions were audio-recorded and transcribed verbatim. Data were coded and organized around identified themes and sub-themes. Working theories were developed by the three authors based on relevant topic characteristics associated with the means in which pharmacists prioritize and choose adverse effect information to communicate to patients.

**Findings:**

Nine pharmacists participated in the discussion. The specific adverse effects prioritized were consistent with the reported highest prevalence. Concepts and connections to three main themes described how pharmacists further tailored patient counseling: potential adverse effects and their perceived importance; patient encounter; and cultural factors. Pharmacists relied on initial patient dialogue to judge an individual’s needs and capabilities to digest safety information, and drew heavily upon experience with other counseling encounters to further prioritize this information, processes dependent upon development and accessing exemplar cases.

**Originality/value:**

The findings underscore practical experience as a critical instructional element of undergraduate health professional patient safety curricula and for developing associated clinical reasoning.

## Introduction

The importance of structured practical experience (clinical internship) in the health professional curriculum, to consolidate, reinforce and advance knowledge and skills, cannot be understated as a critical quality learning determinant and a subsequent comfort as a practicing clinician (Miettinen, 2000; Kolb, 2014). Indeed, it is within authentic care settings, under clinical supervisor guidance, that students participate in patient care experiences to develop and ultimately achieve the professional competencies necessary to work safely and independently (Kogan *et al.*, 2014). Such supervised experiential training assumes importance beyond educational interests and notably includes public health benefits. Medical error is recognized as a leading contributor to healthcare-related morbidity and mortality worldwide, prompting patient safety concepts and practices to become increasingly explicit in health professional education (Makary and Daniel, 2016). In 2011, the World Health Organization (2011) released a multi-professional patient safety curriculum, which recommended specific patient safety principles in course content. Given that medication misadventure, including prescribing errors and adverse drug events, accounts for a sizable proportion of overall medical errors and is often preventable. Unsurprisingly, medication safety principles are, therefore, highlighted in the education of disciplines participating in the drug use process (Frank, 2005; American Association of Colleges of Nursing, 2006). Pharmacy students are trained to ensure drug therapy accuracy and quality and to evaluate medication and patient safety during treatment (Association of Faculties of Pharmacy of Canada, 2010).

When a patient is prescribed or self-selects over-the-counter drug therapy, one pharmacist responsibility is to provide medication counseling, which includes education on how the patient might identify and subsequently manage adverse events. Preventable drug-related emergency department visits and hospital admission have been attributed to inappropriate drug monitoring or failure to evaluate predictable medication side effects, underscoring the need for such dialogue (Beijer and de Blaey, 2002; Forster *et al.*, 2003; Howard *et al.*, 2006). Research demonstrates the high value various patient populations place on such information (Astrom *et al.*, 2000; Nair *et al.*, 2002; Berry *et al.*, 1997; Al Amri, 2009; Piredda *et al.*, 2008; Al-Saffar *et al.*, 2008). However, knowledge gaps persist in studies exploring communicated and retained patient information, up to 60 percent were unaware of safety information associated with their prescribed drugs (Tarn, *et al.*, 2006; Erikson, 2016).

To develop professional competency, pharmacy students must first identify what possible adverse effects are ascribed to drug therapy. This information is readily available from curricular and external resources. Unfortunately, for various medico-legal and academic reasons, the possible adverse effects that may be recorded for any given drug are numerous and include rare, unusual and unlikely reactions. While patients have the right to learn drug therapy risks, it is essential for pharmacists to offer this information within a clinically relevant framework to avoid alarming the patient unnecessarily or unintentionally and introducing discordance with what information the prescriber has previously offered (Jones *et al.*, 2006; Taylor, 2011). Additionally, pharmacists often have little time to offer medication and health counseling during the patient visit. Pharmacy students must then learn to offer accurate and relevant patient-specific education regarding medication risks in an efficient manner. It is still unclear how novice practitioners do so and how undergraduate pharmacy training can improve these processes. Deciding how to effectively teach clinical skills associated with risk communication have been less well explored when compared to diagnostic reasoning and decision-making studies among physicians and medical trainees (Boshuizen and Schmidt, 1992; Norman, 2005; Schmidt and Rikers, 2007; Durning *et al.*, 2013; Bowen, 2006). Little pedagogy extends to the important clinical pharmacy skill: communicating clinically relevant adverse drug effect information to patients. Our objective, therefore, is to: explore how experienced pharmacists identify, prioritize and communicate adverse drug effect information to their patients; and inform pharmacy student training in this regard.

## Methods

A qualitative design employing focus group and semi-structured discussion were used for data collection. In total, 17 pharmacists working in the Heart Hospital, the only specialty cardiology referral center in Qatar, were invited to participate. Previous studies found that approximately 10 percent of patients experience a medication-related adverse event within one month following hospital discharge (Forster *et al.*, 2003). As cardiovascular therapies represent one main drug class implicated in medication-related adverse events, we targeted our inquiry on care providers for this drugs group (Howard *et al.*, 2006). A topic guide was developed following a comprehensive literature review of other quantitative or qualitative assessments that reported side effect counseling or risk communication strategies. Our framework explored consenting participants’ views, experiences and approaches to educating patients regarding treatment safety and tolerability. Selected medications were associated with hypertension, heart failure and myocardial infarction management: hydrochlorothiazide (diuretic); ramipril (angiotensin converting enzyme inhibitor); atenolol (beta blocker); ASA and copylores (antiplatelets). After each discussion, participants were given the opportunity to ask additional questions or make further contributions. The audio-taped focus group and semi-structured interviews were conducted and transcribed by the primary investigator and subsequently verified independently by two research assistants.

Qualitative data analysis was supported by NVivo10^©^ software. Transcripts were read several times by primary investigators to sense the whole and then subjected to latent content analysis. The text was divided into related words, sentences or paragraphs through their content and context as units of meaning. Data were then coded and organized around identified themes and sub-themes based on their similarities and differences. Working theories were drawn by the three primary investigators based on relevant topic characteristics associated with how pharmacists prioritize and choose adverse effect information for communication to patients. Additionally, cardiovascular drug adverse events were documented from electronic tertiary and associated primary resources (Lexicomp© and Micromedex©) for comparison with those side effects and intolerances selected by our participants (Crommelinck and Anseel, 2013; Pelgrim *et al.*, 2013). Ethical approval was obtained from Qatar University Institutional Review Board.

## Results

Nine consenting pharmacists joined the focus group discussion. The other eight were unavailable for a second focus group or did not consent. Specialization in cardiology care averaged six years (sod=2.1). Over 50 intolerances and toxicities are listed in medication product monographs chosen for review; however, the cardiovascular therapy-specific adverse effects prioritized by the participants were mostly consistent with reported highest prevalence (Table I).

Concepts and connections to three main themes were determined, describing how pharmacists tailored patient counseling on potential adverse effects and their perceived importance; encounter; and cultural factors.

### Adverse effects

As indicated, pharmacists prioritized the specific adverse effects they communicated to patients using event prevalence for the drug/s in question. Those with additional perceived importance often accompanied these common adverse effects, namely, ones with life-threatening consequences. The important serious reactions were informed by written resources, influenced heavily by professional experience, e.g., two pharmacists were familiar with hemorrhagic retinopathy occurring during antiplatelet treatment and are now particular about including vision changes that patient’s may detect (a bleeding side effect). Others described how nearly every beta blocker user was reporting fatigue and so they purposefully prepare their patients on what to expect, even before counseling related to low blood pressure or heart rate. Concurrent drug therapy and known concomitant disease states were also invoked as meaningful elements to individualize patient counseling, e.g., highlighting possible shortness of breath in individuals with asthma and prescribed ASA. When asked about their approach to establishing newly marketed medication adverse effects, pharmacists cited electronic monographs (e.g. Lexicomp^©^ or Micromedex^©^) for the usual ones and primary literature resources and government (USA, Europe) health agency safety alerts for those previously underestimated or unanticipated.

### Patient encounter

Significant consideration for patient factors further directed patient education. In addition to confirming or gathering additional information regarding the patient’s medical condition and other medications in the clinical encounter, pharmacists also try to discern other pertinent aspects, such as education level or attitudes toward treatment. They rely on this initial dialogue to judge the individual’s needs and capabilities to digest safety information:

I think first thing for the side effects, you will assess the patient psychologically. So, once you know his condition and once you know how he will accept the information, you will go further or you will stop, you will speak in general speech or you will speak in detail. Sometimes the patients [persist in asking what [are] the side effects? [In these cases] you will go further and tell him [all side effects] even if it’s low percentage.

You should evaluate every person. If he is frustrated […] and he seems not compliant and he is not willing to take the medication, try to avoid that in the end [too much side effect information]. At the same time, because it is your responsibility to state the side effects, just tell them if you find anything that is not like something that is not normal come back to us, talk to us.

Patient encounter length and timing also dictated how side effects were communicated. Outpatient pharmacists described the constraints (practice and patient based) on counseling time owing to volume and occasionally, an individual’s impatience when collecting prescriptions. Conversely, it was acknowledged that this setting could facilitate an ultimately complete and thorough medication education across further follow-up encounters for care and or repeat visits for prescription renewal.

### Cultural factors

Pharmacists understood the social context in which they provide healthcare, weighing professional responsibilities, to give patients precautionary information faced with the populations’ anticipated responses and behaviors:

Shall we speak with the patient frankly and tell him all the side effects expected or do we minimize the side effects, which we can tell the patient? This is the question. We have a different categorization of patients here, especially Qatari or especially Gulf people, we have a good experience with that. If I tell him every side effect, I know about the medication, we have a problem.

If you tell a patient about this [side effects], he might not take the medication because he worries about the side effects, especially in our culture.

Participants pointed out that they must often overcome language barriers when offering even basic directions for safe medication administration. Even when pharmacist and patient share a first language, low literacy levels also posed problems in achieving a dialogue beyond dosing instructions:

[Most] patients, they are not speaking English [or Arabic] by the way. We had a study […] and [most] patients, almost 75%, they are from South Asia so this is one of the [biggest] obstacles for us. So sometimes you have this hard time […] due to the language barrier or due to [poor] literacy also because they are not that educated.

When asked at the focus group’s conclusion for any advice when teaching students about communicating clinically relevant side effects, pharmacists described tactics consistent with some current instructional methods. Adverse effect information is ideally not offered in isolation, but coupled with the potential management strategies and expected responses:

[For ACE inhibitor] If you experience dry cough, then you might want to change the medication, and I always relate the solution to the problem.

[For BB] it is ok to try to decrease exercise in the first week because you may not feel as able as before, but after one week or two weeks you might be able to tolerate [activity].

While our research question and topic guide focused on medication counseling from a safety perspective, pharmacists also stressed that it is drug therapy benefits that are best emphasized during the patient encounter. Finally, they cautioned against a rigid approach to teaching students, instead outlining general processes to maintain flexibility according to each situation.

## Research implications and limitations

We found that expert cardiology pharmacists used widely accessible resources when choosing the most commonly reported adverse effects to communicate during medication counseling, but drew heavily upon other professional judgments to further prioritize this information. Patient experiences reinforced the desire to communicate safety information acquired through other objective means, including high prevalent adverse effects (e.g. gastro-intestinal bleeding and bruising) and some unorthodox ones (e.g. retinal hemorrhage). Some clinicians and educators might dispute the value of addressing unusual side effects, but rare adverse drug reactions (ADRs) may be more relevant in certain settings. Global information related to ADRs arises predominantly from western nations (Ferner and Aronson, 2006). Cultural aspects related to genetic make-up, dietary patterns and alternate available imported or manufactured brands, all influence local medication safety advice and decision making (Wilbur, 2013). Recognizing otherwise obscure side effects in patients could serve as important context-specific pharmacovigilance signal detection if appropriately channeled (Isah *et al.*, 2012; Vaidya *et al.*, 2010; Swamy *et al.*, 2015).

There is little about clinical reasoning (clinical decision making or clinical judgment) found in the pharmacy literature (Gregory *et al.*, 2016). Existing biomedical research pertains to nurses, therapists and physicians, namely, distinguishing how novice and experts arrive at initial diagnosis following patient history and physical examination data gathering (through actual or simulated means) (Field, 2004; Jensen *et al.*, 2000; Ericsson, 2007; Daley, 1999). However, consistent with various clinical reasoning models, our findings do not suggest discipline-distinct processes when determining patient-specific risk communication decisions (Marcum, 2012). The way pharmacists prioritized specific drug safety elements for patient counseling was often affected by prior experiences. As previously determined in other professions, clinicians store and organize information related to patient conditions in their memory as an illness script and can then draw upon these models to inform care decisions (Charlin *et al.*, 2007). As it pertains to medication counseling, pharmacists would not use such exemplars for medical diagnostic purposes *per se*, but instead to recognize patient dispositions. Verbal and non-verbal cues related to language, education, engagement and the individual’s affect may be discerned to guide re-framing side effect information to optimize understanding, given previous counseling outcomes with similar individuals. Such patient-tailored messaging is meaningful. Studying patient preferences and the way they understand written and verbal medication safety information shows that information is not uniformly interpreted and can have negative implications on medication use (Tong *et al.*, 2016; Berry *et al.*, 1998). Unintentionally provoking therapeutic non-adherence by adverse effect counseling is a well-documented concern among health providers in the region (Wilbur *et al.*, 2015; Silbernann and Hassan, 2011; Al Qasem *et al.*, 2011). As in some Asian, European and Latin countries, non-disclosure is prevalent among Arab Middle Eastern cultures (Faria and Southami, 1997). Communication style for Arabic speakers may be indirect in that facts or ideas that might prove distressing are not explicated (Feghali, 1997). Fear about medication side effects has not been identified as a patient-related factor for non-adherence in the region (Al Qasem *et al.*, 2011). Indeed, patient studies in the Middle East indicate preference for such information (Al-Saffar, *et al.*, 2008).

Like other clinical reasoning applications, such as arriving at a diagnosis and choosing a management plan, the risk communication strategy may be challenging to replicate in undergraduate health professional curricula (Han *et al.*, 2014; Thistlethwaite and Tie, 2007). For pharmacy students, side effect mechanisms and prevalence may be covered in pharmacology and therapeutics courses, respectively, and may even be noted in critical appraisal exercises using evidence-based medicine. Meanwhile, communicating ADR information may be practiced with peers or standardized patients in professional skills courses (Mesquita *et al.*, 2010). While experienced clinicians make decisions according to deliberate analysis, concurrent complementary pattern recognition of illness scripts is not necessarily a conscious process and therefore not easily taught (Ericsson, 2007). Campus-based strategies to scaffold pre-existing knowledge, for use in patient-case solving, include problem-based learning, self-explanations and schematically representing ideas (concept maps) (Bowen, 2006). However, mastering theoretical or simulated medication and patient safety content and communication does not guarantee translation into professional competency within diverse care settings (Rudaz *et al.*, 2013). Experiential learning remains paramount for health professional students to build illness script databases enriched with repeated exposure to patients over time and the deliberate practice of risk communication guided by frequent feedback (Ericsson, 2008). Pharmacy students enrolled in North-American accredited programs will complete up to 40 weeks in direct-patient care settings before graduation (Accreditation Council of Pharmacy Education, 2016; Canadian Council for Accreditation of Pharmacy Programs, 2014). Clinical supervisors can actively translate clinical concepts into safe patient care through purposeful questioning, by encouraging reading prototypical cases for comparison with encountered patients and employing think-aloud techniques to further support connections between patient encounters (Bowen, 2006; Lubarsky *et al.*, 2015). Finally, our participants encouraged student abilities consistent with clinical reasoning skills, namely, tolerance for ambiguous situations that they will assuredly face in practice.

While our study is the first to explore how pharmacists make decisions about communicating safety information during medication counseling, several limitations are noted. Participants were sampled from healthcare providers specializing in cardiology. We do not know how experts in other medical disciplines make decisions regarding medication risk communication. However, studying nurses and pharmacists working locally in oncology settings also identify that clinician assessment of the patient’s psychological health as a consideration in side effect education (Wilbur *et al.*, 2015). Our findings may also be context specific to the Middle East. Qualitative studies are unlike quantitative inquiries, in that generalizability is theoretical as opposed to statistical, but pharmacists providing medication counseling in cultural settings where patients expect that adverse effect disclosure may use different approaches to prioritizing safety information. We used focus group discussions to answer our research question, but ethnographic methods, such as directly observing patient counseling encounters, could have yielded actual risk communication dialogue that is different from what was described by pharmacists. Similarly, we might have also observed our pharmacists supervising students to determine what strategies they use to role model side effect counseling and the feedback given to students interacting with patients.

## Conclusions

Pharmacist decision making, when prioritizing medication risk communication, relied heavily on prior patient encounters. Practical experience as a critical undergraduate health professional patient safety instructional element is therefore underscored. Strategies for how clinical supervisors can develop associated clinical reasoning should be formally promoted in pharmacy programs.

## Figures and Tables

**Table I:** Adverse effects identified by pharmacists

Drug therapy	Pharmacist prioritization	Prevalence (%)^a^
Thiazide-like diuretic: hydrochlorothiazide	Hypokalemia	15-50
	Hyperglycemia	1-5
	Hyperuricemia	1-3
ACE inhibitor: ramipril	Hyperkalemia	1-10
	Dry cough	8-12
	Hypotension	11
	Renal artery stenosis	1
Beta blocker: atenolol	Fatigue	1-10
	Hypotension	1-27
	Bradycardia	1-16
Antiplatelet	Minor bleeding	3-5.6
	Pruritis (cladogram)	2-4
	Allergy	4-23
	Gastric upset (aspirin)	6-31

**Sources:** ^a^Lexicomp Online Database (2016), Lexicomp Inc. (http://online.lexi.com/, accessed July 20, 2016); Micromedex Drugdex (2016), Truven Health Analytics (www.micromedexsolutions.com/, accessed July 20, 2016)
